# UNDERGRADUATE STUDENTS CONDUCTING GERONTOLOGY RESEARCH: AN AVENUE FOR INTERGENERATIONAL CONNECTION

**DOI:** 10.1093/geroni/igae098.0182

**Published:** 2024-12-31

**Authors:** Abigail Stephan, Vanessa Martinez, Daniel Moss, Mary Walton, Ava McVey, Kalvry Cooper, Christine Phillips, Lesley Ross

**Affiliations:** Clemson University, Clemson, South Carolina, United States; Clemson University, Clemson, South Carolina, United States; Clemson University, Clemson, South Carolina, United States; Furman University, Greenville, South Carolina, United States; Clemson University, Clemson, South Carolina, United States; Clemson University, Clemson, South Carolina, United States; Clemson University Institute for Engaged Aging, Seneca, South Carolina, United States; Clemson University, Clemson, South Carolina, United States

## Abstract

With the proportion of older adults and subsequent rates of age-related assets (e.g., wisdom) and challenges (e.g., Alzheimer’s disease/related dementias), projected to rise over the coming decades, it is imperative that professionals across disciplines understand how physical, cognitive, and everyday function in older adulthood impact individuals, families, and communities. As educators, we have a responsibility to train professionals to be competent and aware of older adults’ needs, whether or not students plan to pursue careers directly related to gerontology or geriatrics. Many forms of experiential learning have been employed to accomplish this goal, though students’ direct involvement in gerontology research has not been examined in detail. Our investigation debriefs the experiences of students and faculty involved in a pilot gerontology study where undergraduate students conducted in-person visits with older adult participants. Feedback was collected through anonymous online surveys, guided focus groups, and unstructured team conversations. A salient theme, and the focus of this presentation, was meaningful engagement between students and study participants. Students described gaining personal insights and professional skills from working with participants. Notably, we found that several structures supported positive connections (e.g., in-person interactions, pairing two student testers with a participant, shared goals, balance of structured activities and wait time for conversation). Areas for improvement included additional training for managing participants’ study task pacing. Although these findings reflect lessons learned from one case, they can guide future endeavors of faculty who want to include undergraduate students in their research while promoting intergenerational learning.

